# Solitary Necrotic Nodules of the Liver: Histology and Diagnosis With CT and MRI

**DOI:** 10.5812/hepatmon.6212

**Published:** 2012-08-20

**Authors:** Li Xia Wang, Kan Liu, Guang Wu Lin, Ren You Zhai

**Affiliations:** 1 Department of Radiology, Beijing Chaoyang Hospital, Capital University of Medical Sciences, Beijing, China; 2 Department of Radiology, Cancer Hospital, Chinese Academy of Medical Sciences, Peking Union Medical College, Beijing, China; 3 Department of Radiology, Huadong Hospital, Fudan University, Shanghai, China

**Keywords:** Liver Neoplasms, Tomography, X-Ray Computed, Magnetic Resonance Imaging

## Abstract

**Background:**

A solitary necrotic nodule (SNN) of the liver is an uncommon lesion, which is different from primary and metastatic liver cancers.

**Objectives:**

To analyze the classification, CT and MR manifestation, and the pathological basis of solitary necrotic nodule of the liver (SNN) in order to evaluate CT and MRI as a diagnosing tool.

**Patients and Methods:**

This study included 29 patients with liver SNNs, out of which 14 had no clinical symptoms and were discovered by routine ultrasound examinations, six were found by computed tomography (CT) due to abdominal illness, four had ovarian tumors, and five had gastrointestinal cancer surgeries, previously. Histologically, these SNNs can be divided into three subtypes, i.e., type I, pure coagulation necrosis (14 cases); type II, coagulation necrosis mixed with liquefaction necrosis (five cases); and type III, multi-nodular fusion (10 cases). CT and magnetic resonance imaging (MRI) patterns were shown to be associated with SNN histology. All patients were treated surgically with good prognosis.

**Results:**

CT and MRI appearance and correlation with pathology types: three subtypes of lesions were hypo-density on both pre contrast and post contrast CT, 12 lesions were found the enhanced capsule and 1 lesion of multi- nodular fusion type showed septa enhancement. The lesions were hypo-intensity on T2WI and the lesions of type II showed as mixed hyperintensity on T2WI. The capsule showed delayed enhancement in all cases, and all lesions of multi- nodular fusion type showed delayed septa enhancement on MR images. 15 cases on CT were misdiagnosed and Four cases on MRI were misdiagnosed and the accuracy of CT and MRI were 48.3% and 86.2% respectively.

**Conclusions:**

In conclusion, CT and MRI are useful tools for SNN diagnosis.

## 1. Background

Solitary necrotic nodules (SNNs) of the liver are uncommon lesions that were first found in 1983 by Shepherd and Lee (1). An SNN is a small sub-capsular nodule containing a necrotic core surrounded by collagenal fibrous tissues. Most cases were asymptomatic and were detected by preoperative evaluation for another cause or incidentally during surgery. Indeed, a previous study (2) reported one case in which the lesion subsequently showed complete regression over time. Thus, conservative treatment and management with close follow-up may be considered as a good treatment option for SNNs (3, 4). However, studies to date have also found that SNNs could contain foci of metastatic cancer cells that are mainly associated with a gastrointestinal malignancy (5), SNN etiology may include trauma, parasitic infection (6), and sclerosing hemangioma (7, 8). In any event, detection of SNNs in the liver has significantly increased with the evolution of imaging technology. Dynamic three-phased enhanced computed tomography (CT) has improved the detection and characterization of liver neoplasms from SNNs (9, 10, 11). Although preoperative ultrasonography (US) and CT features have been reported previously in SNNs (12), MRI has been used to provide accurate information regarding the diagnosis of SNNs and discriminate this entity from metastatic liver tumors and intrahepatic cholangio-carcinoma (13). However, due to a large amount of coagulated necrotic cellular material within the tumor, biopsies of these lesions are frequently misinterpreted as metastases or primary liver tumors.

## 2. Objectives

In this study, 29 cases of SNNs were reported; their histology types, also their CT, and MRI features were described; and their CT and MRI data were correlated with pathology results in order to differentially diagnose SNNs from other nodules found in the clinic.

## 3. Patients and Methods

### 3.1. Study Population 

Between May 2004 and March 2011, 29 patients with SNNs were admitted to three medical centers (i.e., Beijing ChaoYang Hospital of Capital University of Medical Sciences, Cancer Hospital of the Chinese Academy of Medical and Sciences, and HuaDong Hospital of Fudan University). All patients underwent surgery, and the SNNs were pathologically confirmed. These patients included 18 (62 %) males and 11 (38 %) females with a median age of 51 years old, ranging between 30 and 70 years old. Of these 29 patients, 14 had no clinical symptoms and their problems were found by routine ultrasound examinations, problems in six other patients were found by CT due to abdominal discomforts (i.e., two gastritis, two appendicitis, one gallstone, and one kidney stone), four had ovarian tumors (two serous cyst adenoma, one mucinous cyst-adenoma, and one cyst adeno-carcinoma), and five had gastrointestinal cancer surgery previously. These 29 patients had normal liver function and normal serum levels of alpha-fetoprotein (less than 400 ng/ml) and blood leukocyte counts, but they were negative for the viral hepatitis B antigen. Inclusion and exclusion criteria were as follows: only those patients who underwent pre-and post-enhanced CT and MRI were included, whereas those who did not undergo liver biopsy or surgery were excluded from this study. All patients underwent CT and MRI and signed a written informed consent form. This study was approved by the institutional review board.

### 3.2. CT and MRI Analysis (Helical CT) 

All 29 patients underwent abdominal helical CT, which included pre-enhanced and post-enhanced imaging. All CT examinations were performed by multi-detector row scanners (SOMATOM Definition, Siemens Medical Systems, Erlangen, Germany; Light Speed Ultra, GE Healthcare, Milwaukee, WI). Patients were fasting Six hours before CT examination. The patients were in the supine position, the scanning range was from the right diaphragm to the kidney, the slice thickness was 1.25 millimeters, and the reconstruction interval was five millimeters with a pitch of 0.875:1. All patients also received nonionic intravenous contrast material (Ultravist, Bayer Technology and Engineering Company Limited, Berlin, Germany), which was administered at a rate of 3 mL/sec (120–150 mL total volume) by a mechanical power injector (Medrad, Pittsburgh, PA, USA) set at 180–250 mAs and 120 kVp. Thereafter, the images were taken with hepatic arterial, portal venous phase, and delayed-phase imaging with delays of 25 sec, 50–60 sec, and 2–3 min, respectively.

### 3.3. MRI Examination 

In addition, 17 patients underwent MRI using a 3.0-T system (Signa Horizon HD 3.0T, GE Healthcare, and Milwaukee, Wis). MRI was performed with a variety of software upgrades that continuously evolved during the study period. Sequences included T2-weighted fast spin echo with fat-suppression techniques [repetition time (TR, msec)/echo time (TE, msec), 4781/81; field of view, 38 cm; matrix, 256 × 256; number of sections, 20; section thickness, 8 mm; and one signal acquired] and T1-weighted in-phase and opposed-phase gradient echo (TR/TE, 210/2.45, 210/4.72; flip angle, 80°; field of view, 38 cm; matrix, 256 × 256; number of sections, 20; section thickness, 8 mm; and one signal acquired). LAVA imaging was also performed in all 17 patients after contrast material administration (a dose of 0.1 mmol per kg of body weight and 3–5 mL/ sec, followed by a 20 mL saline flush) during the hepatic arterial (20–25 sec delay) and portal venous (60–70 sec delay) phases. Delayed phase imaging (3-5 min after contrast material injection) was performed in all patients. 12 patients were examined in a 3.0-T system (Magnetom, Siemens Medical Systems, and Erlangen, Germany) by a phased-array torso coil for signal reception. A breath-hold T1-weighted fast field-echo pulse sequence was performed in all patients with a chemical shift technique (also called in-phase and opposed-phase imaging with fat-suppressed T1-weighted MR sequences. The parameters were set as TR/TE (msec), 210/2.45, 210/3.83; flip angle, 65°; field of view, 38 cm; matrix, 256 × 256; number of sections, 20; section thickness, 8 mm; and one signal acquired. A respiratory-triggered T2-weighted fast spin-echo pulse sequence was also performed. The acquisition parameters were as follows: TR/TE (msec), 4781/81; flip angle, 140°; field of view, 38 centimeter; matrix, 256×256; number of sections, 20; section thickness, 8 millimeter; and one signal acquired. In these patients, a T1-weighted gradient-echo MR sequence (VIBE) was performed during the hepatic arterial, portal venous, and delayed phases after administration of gadolinium-based contrast medium at a dose of 0.1 mmol/kg and 3–5 mL/sec, followed by a 20 mL saline flush.

### 3.4. Image Analysis 

All images were retrospectively and blindly analyzed by two experienced radiologists who did not know the pathology data. If the results were inconsistent, they discussed the images again and obtained unanimous views.

## 4. Results

### 4.1. Histology

**Figure 1:**
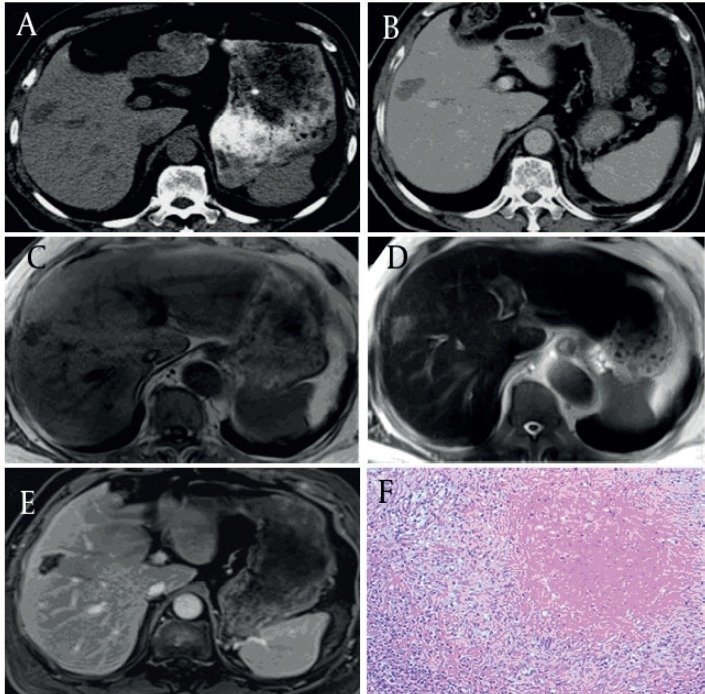
Pure Coagulation Necrosis Type of Solitary Necrotic Nodules (SNN) in a 45-Year-Old Woman (black arrow) (A) Pre-contrast CT image of the right liver lobe; the SNN is a hypo-attenuated mass compared with the liver parenchyma. (B) Post-contrast CT image at the portal venous phase; the lesion shows no enhancement except for mild peripheral capsule enhancement. (C) By transverse T1WI, the SNN is hypo-intense relative to the liver parenchyma. (D) By transverse T2WI, the SNN is slightly hyper-intense relative to the liver parenchyma. (E) Contrast-enhanced T1WI at the portal venous phase; it shows no enhancement of the lesion except for moderate peripheral capsule enhancement. (F) Histology of the liver biopsy sample showed central necrotic tissue surrounded by a borderline zone containing infiltrating inflammatory cells and fibrotic changes. The outer layer shows normal hepatocytes (hematoxylin-eosin stained section, original magnification x100).

**Figure 2:**
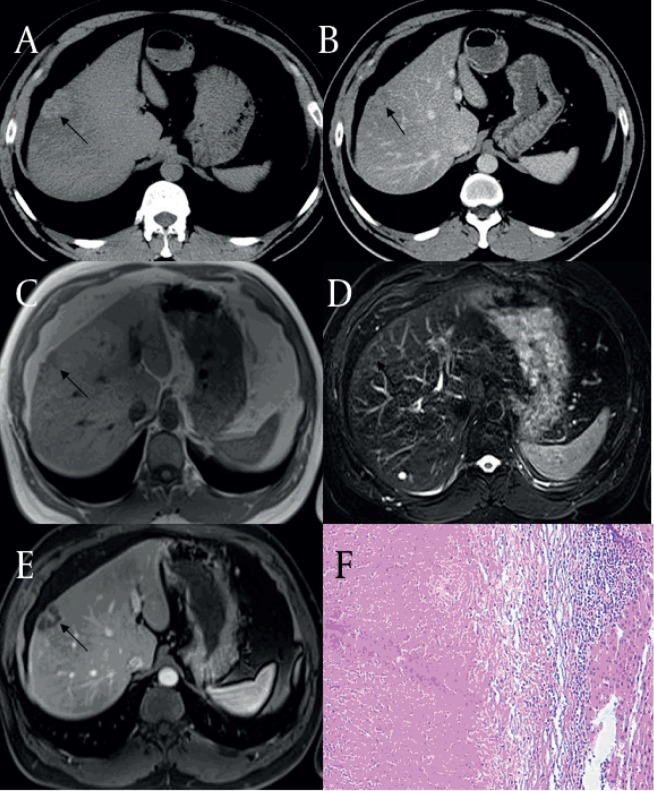
Coagulation Necrosis Mixed With a Liquefaction Necrosis Type of Solitary Necrotic Nodules (SNN) in a 50-Year-Old Man (GE Medical System) (black arrow) (A) By transverse T1WI, the SNN is heterogeneous hypo-intensity relative to liver parenchyma at the right lobe of the liver. (B) By transverse T2WI, the SNN is slightly hyper-intense mixed with hyper-intense foci (white head arrow) relative to the liver parenchyma. Contrast-enhanced T1WI at arterial phase (C), at portal venous phase (D) and coronal contrast-enhanced T1WI at delay phase (E) shows no enhancement of the lesion except for marked peripheral capsule enhancement. (F) Histology of the SNN shows the liquefaction necrosis core surrounded by a coagulation necrosis area and infiltrating inflammatory cells (Hematoxylin-eosin stain; original magnification x100).

**Figure 3:**
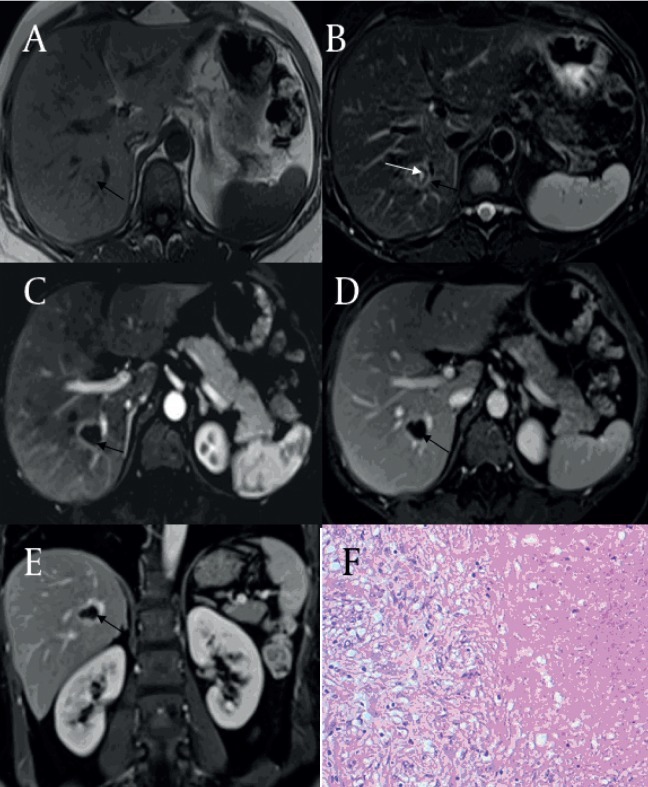
Multi-Nodular Fusion Type of Solitary Necrotic Nodules (SNN) (Black Arrow) in a 65-Year-Old Woman A) On pre-contrast CT image, SNN is hyper-attenuating mass relatively to the background of fatty liver parenchyma. (B) By post-contrast CT imaging at the portal venous phase, the lesion shows no enhancement and an invisible peripheral capsule. On in phase (C) T1WI, the SNN is hypo-intensity relatively to liver parenchyma at the right lobe of the liver. (D) On T2WI, the SNN is isointense relative to liver parenchyma. (E) Contrast-enhanced T1WI at the delayed phase shows no enhancement of the lesion except at the septa and peripheral capsule enhancement. (F) Histology of the SNN shows multiple fusion nodules, and most lesions had coagulation necrosis surrounded by a fine fibrous capsule with infiltrating lymphocytes, plasma cells, and eosinophilic granulocytes (Hematoxylin-eosin stain; original magnification, x100).

All 29 patients had a single lesion in the liver, 23 of which were localized in the right lobe and six in the left lobe. The mean diameter was 3.0 centimeters, ranging from 1.0 to 4.7 cm. Macroscopically, the color of the nodules (lesions) was gray-yellow; and microscopically, 14 lesions were pure coagulation necrosis, five cases were mixed with liquefaction necrosis in the comparatively large nodules, and 10 lesions were multi-nodular fusions (six lesions had crack-like necrosis). All the lesions had peripheral hyaline fibrosis capsules and infiltration of lymphocytes, plasma cells, eosinophilia granulocytes, and a few leukocytes. The surrounding liver tissues had a normal appearance without cirrhosis in 28 cases, while one case showed fat-degeneration in the surrounding liver tissues. The bacteriological examination and acid-fast staining results were all negative. According to the pathology reports, these SNNs were divided into three subtypes, i.e., type I, pure coagulation necrosis (14 cases; Figure 1F); type II, coagulation necrosis mixed with liquefaction necrosis (five cases; Figure 2F); and type III, multinodular fusions (10 cases; Figure 3F).

### 4.2. CT Diagnosis 

By conventional CT, 22 lesions were localized at the superficial liver (the distance to the liver capsule was less than 2 cm, 18 were in the right lobe, and four were in the left lobe), and seven lesions were localized in deep lobes (the distance to the liver capsule was more than 2 cm, five were in the right lobe, and two were in the left lobe). The diameter of these 29 lesions was between 0.8 and 5.0 cm. The lesions had clear boundaries and were slightly hypo-dense or iso-dense relative to the density of the liver. Not all lesions were enhanced on the post-enhanced CT scan. 12 lesions had a thin capsule and were mild or moderately enhanced.

### 4.3. MRI Diagnosis 

By MRI, 29 lesions showed that the diameter of 15 round-like lesions was 0.8–3.6 cm andthat the diameter of 14 irregular lesions was 1.7–4.7 centimeters. The lesions had clear boundaries and were slightly hypo-intense compared with the signal intensity of the liver by T1-weighted imaging. All lesions were iso-intense or hypo-intense by T2-weighted imaging, and 11 lesions were dot-like or patch-like hyper-intense (diameter of six lesions was greater than 3; 10 lesions had internal septa and appeared as a multi-nodular fusion). Not all lesions were enhanced on a dynamic scan after Gd-DTPA administration; in the portal phase, the lesion boundary was clear relative to the liver parenchyma. All 29 lesions had a thin capsule and were mild or moderately enhanced by dynamic T1WI and delayed-phase T1WI. 

### 4.4. Association of Histology With CT and MRI Data 

Association of the SNN pathology results with the CT and MRI data are reported in Tables 1 and 2 and in Figures 1, 2 and 3. Lesions and capsules were often found (12/29, 41.4 %) by post-enhanced CT; but the inner-structures such as liquefaction necrosis and septa were not easily found (1/10, 10 %) by CT scan. The hypo-intensities shown by T1WI and T2WI were associated with coagulation necrosis in the lesions, and the hyper-intensity shown by T2WI was associated with liquefaction necrosis. The capsules and septa were shown on post-enhanced MRI, especially in the delayed phase. 11 lesions were found to be mixed with liquefaction necrosis (five cases of type II and six cases of type III), which was confirmed by histology. T2-weighted imaging could show liquefaction necrosis within the lesions, but CT did not find liquefaction necrosis. These 29 lesions were found to have an enhanced capsule on the delayed phase of the enhanced MRI, but only 12 lesions were found to have an enhanced capsule by enhanced CT. Ten cases had septa within the lesions, and were all found by enhanced MRI, but only one lesion had septa by enhanced CT (Table 3). All 29 lesions were found and confirmed by CT and MRI. Among them, nine cases were misdiagnosed as a metastasized tumor by CT due to the patients’ history of ovarian tumors or gastrointestinal cancer, four lesions were considered as granulomatous lesions, two were considered as cholangio-carcimoma, and the remaining cases were diagnosed as SNNs. By MRI, two cases of type II and two cases of type III SNNs were misdiagnosed as metastasized tumors, while the others were diagnosed as SNNs. Combined CT and MRI diagnosed the cases accurately except one case of type II and one case of type III SNNs which were misdiagnosed as metastasized tumors in MRI. All 29 patients underwent liver surgery and had follow-up CT or MRI procedures after their operations. 16 patients were monitored for more than five years, and no recurrence was found.

**Table 1:** Association of Solitary Necrotic Nodules (SNN) Histology With Comuted Tomography Patterns

	** Patients, No.**	**Unenhanced CT**			**Enhanced CT**		
		**Hypo density**	**Iso-density**	**Hyper-density**	**Unenhanced**	**Septa**	**Capsule**
**I**	14	11	3	0	10	0	4
**II**	5	3	2	0	3	0	2
**II**	10	9	0	1	3	1	6

Abbreviation: CT, computed tomography

**Table 2:** Association of Solitary Necrotic Nodules (SNN) Histology With MRI Patterns

	**Patients, No.**	**T1WI (SI)**		**T2WI (SI)**			**Dynamic CE T1WI**			**Delayed CE T1WI**		
		**Hypo**	**Mix**	**Hypo**	**Iso**	**Mix-hyper**	**Nono**	**Septa**	**Capsule**	**Nono**	**Septa**	**Capsule**
** I**	14	14	0	6	8	0	2	0	14	2	0	14
** II**	5	4	1	1	0	4	3	0	5	3	0	5
** III**	10	4	6	4	0	6	0	10	10	0	10	10

Abbreviation: SI, signal intensity; CE, contract enhanced

**Table 3:** Solitary Necrotic Nodules (SNN) Appearances in Both Comuted Tomography and Magnetic Resonanse Imaging

	**CT**				**MRI**		
	**Cases, No.**	**Unenhanced CT**	**Enhanced CT**	**Cases**	**T1WI**	**T2WI**	**Enhanced MRI**
** Liquefaction necrosis**	11	0	0	11	7	11	0
** Capsule**	29	0	12	29	0	0	29
** Septa**	10	0	1	10	0	0	10

Abbreviation: CT, computed tomography; MRI, magnetic resonanse imaging

## 5. Discussion

In this study, data belonging to 29 patients with SNNs in the liver were collected and analyzed. Out of these 29 patients, 14 had no clinical symptoms and their problems were discovered through routine ultrasound examinations, problems in six patients were found by CT due to abdominal illness, four had ovarian tumors, and five had previous gastrointestinal cancer surgery. Histologically, these SNNs showed three recognizable patterns, i.e., pure coagulation necrosis, coagulation necrosis mixed with liquefaction necrosis, and multi-nodular fusion. Nine out of these 29 patients were initially misdiagnosed as having a metastasized tumor by CT. All patients were treated surgically with good prognosis. Thus, it can be concluded that CT and MRI are useful tools for SNN diagnosis.

According to the literature to date, an SNN in the liver is an unusual liver lesion and most often occurs in male patients between 50 and 70 years of age. Most SNNs are localized close to the liver surface of the right liver lobe. SNN is usually asymptomatic, and the majority of reported cases have been incidental findings with normal liver function at postmortem, operation, or radiological investigation (4). To date, SNNs have not been found in hepatitis B-infected or cirrhosis patients. In fact the current study clearly confirmed these SNN patterns and also indicated that SNNs are often found as single nodules in the liver and sometimes as multi-nodular fusions. The boundary of the SNN was clear, and liquefaction necrosis frequently occurred. However, the etiology that caused the SNN should be determined, although various hypotheses have been proposed (1, 6, 7, 14, 15). In the current study, no feeding arteries or history of parasitic infections were found. It was proposed that the SNN might have resulted from trauma or an immune response, followed by self-defense to form fibrous tissues surrounding the coagulation necrosis (16, 17, 18). Furthermore, a previous study showed foci of metastatic carcinoma in SNN that was associated with gastrointestinal cancer (5), and included four cases of ovarian tumors and five cases of gastrointestinal cancer. However, this case did not occur in our study. The histopathology of SNN mainly comprised coagulative necrosis surrounded with thin boundary of collagen fibers, scanty mononuclear, lymphocyte, plasmocyte inflammatory cells and elastic fiber, and the central zone had a rough patchy appearance with cellular debris. In addition to the standard histological criteria of solitary necrotic nodules, and the relatively small size (15 mm or less) , the frequent presence of calcifications seem to characterize this enigmatic entity further (19). But calcifications were not found in the CT images of our patients. Tsui WM (6) found that in the two cases where ghosts of degenerated cells and partially preserved liver reticulin pattern were noted, worms were identified. Deniz K (20) thought that some of these lesions were focal infarctions of the liver parenchyma because necrotic hepatocytes with bile pigment and well-preserved reticulin framework were consistent with early hepatic parenchymal infarction. And he thought the mechanism was gastrointestinal malignant tumors that seemed able to affect hepatic microvasculature via the portal vein thus contributing to the development of SNNL. As for clinical SNN diagnosis, CT and MRI are very useful tools that can be associated with the pathological features of SNNs. For example, in the pure coagulation necrosis type of SNN, the lesions usually have a homogeneous density by conventional and enhanced CT; as expected, no enhancement was found in this study. The capsules appeared as delayed enhancement. The current finding is consistent with the literature (18). In the coagulation necrosis mixed with liquefaction necrosis type of SNN, it appeared as iso-intensity mixed with foci or patch-like hyper-intensity by T2WI, and the fibrous capsule appeared as a mild-moderate enhancement. Compared with the pathology results, the hyper-intensity on T2WI was due to liquefaction necrosis and a peripheral area made of coagulation necrosis. If the lesions were larger than 2.5 cm, they appeared as a hyper-intense area by T2WI, and no obvious septa were found; thus, they were defined as type II. Compared with CT and T1WI, T2WI was more sensitive and identified the liquefaction necrosis area more easily. In the multi-nodular fusion type of SNN, the septa and capsules were frequently shown on the MRI with an iso-intense signal by T1WI and T2WI and mild or moderately delayed enhancement after Gd-DTPA administration. Compared to the histology results, the lesions showed multiple fusion nodules, and most of the lesions were coagulated and mixed with patch-like liquefaction necrosis. In addition, it was found that MRI is better than CT to find liquefaction necrosis, septa, and capsules. T2WI could show liquefaction necrosis in all lesions, but CT could not. Capsules were found by MRI in all 29 cases but only in 41.4 % (12/29) of the cases by enhanced CT; and septa were not found by CT at all. By analyzing these misdiagnosed cases, it was found that CT was unable to find any inner liquefaction necrosis and was more likely to misdiagnose an SNN as a metastasized tumor when the lesions had enhanced capsules. Similarly, four patients were misdiagnosed as having metastasized tumors by MRI due to obvious liquefaction necrosis that resulted in hyper-intensity on T2WI and mild boundary enhancement on dynamic enhanced MRI. On MRI the marked necrosis of SNNs which were similar to metastasis were not found in CT, and no enhancement wasfonnd either,the nodule or the boundary, so CT refused the diagnosis of metastasis, and by combined CT and MRI, the accuracy of diagnostic SNNs can be increased. With SNNs in mind, it is relatively easy to make a differential diagnosis of SNN or other lesions, although lack of experience and standard enhanced CT or MRI methods (9, 21, 22) could lead to misdiagnosis.
